# Targeting Aldehyde Dehydrogenases to Eliminate Cancer Stem Cells in Gynecologic Malignancies

**DOI:** 10.3390/cancers12040961

**Published:** 2020-04-13

**Authors:** Vaishnavi Muralikrishnan, Thomas D. Hurley, Kenneth P. Nephew

**Affiliations:** 1 Medical Sciences Program, Indiana University School of Medicine, Bloomington, IN 47405, USA; vmuralik@iu.edu; 2 Department of Biochemistry & Molecular Biology, Indiana University School of Medicine, 635 Barnhill Drive Medical Science, Indianapolis, IN 46202, USA; 3 Department of Anatomy, Cell Biology and Physiology and Obstetrics and Gynecology, Indiana University School of Medicine, Indianapolis, IN 46202, USA; 4 Indiana University Simon Comprehensive Cancer Center, Indianapolis, IN 46202, USA

**Keywords:** gynecologic malignancies, cancer stem cells, aldehyde dehydrogenases

## Abstract

Gynecologic cancers cause over 600,000 deaths annually in women worldwide. The development of chemoresistance after initial rounds of chemotherapy contributes to tumor relapse and death due to gynecologic malignancies. In this regard, cancer stem cells (CSCs), a subpopulation of stem cells with the ability to undergo self-renewal and clonal evolution, play a key role in tumor progression and drug resistance. Aldehyde dehydrogenases (ALDH) are a group of enzymes shown to be robust CSC markers in gynecologic and other malignancies. These enzymes also play functional roles in CSCs, including detoxification of aldehydes, scavenging of reactive oxygen species (ROS), and retinoic acid (RA) signaling, making ALDH an attractive therapeutic target in various clinical scenarios. In this review, we discuss the critical roles of the ALDH in driving stemness in different gynecologic malignancies. We review inhibitors of ALDH, both general and isoform-specific, which have been used to target CSCs in gynecologic cancers. Many of these inhibitors have been shown to be effective in preclinical models of gynecologic malignancies, supporting further development in the clinic. Furthermore, ALDH inhibitors, including 673A and CM037, synergize with chemotherapy to reduce tumor growth. Thus, ALDH-targeted therapies hold promise for improving patient outcomes in gynecologic malignancies.

## 1. Introduction

The first line of therapy for most gynecologic cancers includes surgery, followed by chemotherapy and radiation [1]. However, in the majority of cases, these conventional therapies do not completely eliminate the malignant cells. The primary reason for high mortality is recurrence and subsequent metastasis caused by the residual population of cancer cells [2,3]. The cells that survive after the first line of treatment and contribute to cancer recurrence are known as CSCs [4,5]. The CSC theory states that the tumor is a heterogeneous mass, and within the tumor exists a hierarchy of cells, with CSCs at the apex [6]. Lapidot et al. first proposed the idea that a set of specialized cells present within the tumor can sustain and repopulate the tumor [7]. CSCs have since been reported in gynecologic malignancies (Table 1). 

CSCs are resistant to conventional chemotherapy due to several mechanisms. Chemotherapeutic drugs, primarily platinum-based drugs, form DNA crosslinks, killing cells by causing DNA damage in rapidly-dividing cells [21]. However, CSCs are resistant to DNA damage due to a number of properties, including slow cycling, reduced uptake of drugs and increased drug efflux due to the high expression of a class of non-selective drug transporters called adenosine triphosphate binding cassette (ABC) ATPases [22]. Furthermore, CSCs have enhanced DNA repair due to overexpression of repair pathways such as ataxia-telangiectasia-mutated (ATM), ataxia telangiectasia and rad3-related (ATR), checkpoint kinase 1 (Chk1), poly(ADP-ribose) polymerase 1 (PARP1), and RAD51 [23] that protect CSCs from drugs designed to cause cancer cell death by inducing DNA damage. As a quiescent population [24], CSCs are further protected by platinum-induced DNA damage. Thus, it is necessary to target CSCs specifically to achieve a better prognosis in patients. Of the different CSC markers identified to date in gynecologic malignancies [10,11,12,13,14,15,16,17,18,19,20,22,23], ALDH is widely recognized as a highly robust CSC marker across the vast majority of cancer types, including gynecologic CSCs. Furthermore, ALDH holds the distinction of having potential functional importance in the maintenance of CSCs [25], making it an attractive target for eradicating CSC in the therapeutic maintenance setting for gynecologic malignancies such as ovarian cancer.

**Figure 1:**
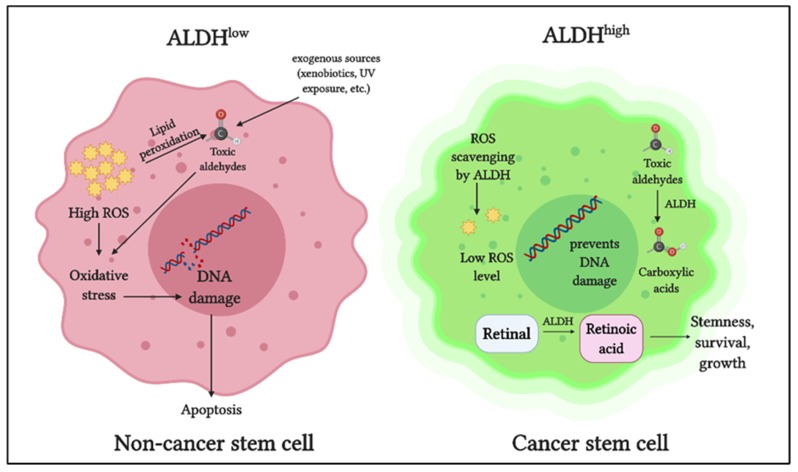
Role of aldehyde dehydrogenases (ALDH) in cancer stem cells: ALDH detoxifies toxic aldehydes (endogenous and exogenous) into less toxic carboxylic acids. ALDH maintains intracellular reactive oxygen species (ROS) at a low level thus preventing oxidative stress and DNA damage. ALDH oxidizes retinaldehyde into retinoic acid, which promotes stemness, growth, and survival in cancer stem cells.

The ALDH superfamily comprises 19 members, all of which are involved in regulating crucial functions in normal as well as cancer stem cells [13,14,15,16,17,18,19]. The primary role of ALDH enzymes is to metabolize reactive aldehydes produced by various biological processes [26] (Figure 1).

Detoxification of aldehydes is critical for cellular health, as aldehyde toxicity can lead to DNA damage, impaired cellular homeostasis, and cell death [27]. Another vital role of ALDH is in retinoic acid metabolism, which is crucial for gene expression and morphogenesis during embryonic development growth, cellular differentiation, and homeostasis of vertebrates [28,29,30]. Cytosolic class I ALDH enzymes catalyze the NAD-dependent oxidation of both all-trans-retinal and 9-cis-retinal to all-trans-retinoic acid and 9-cis-retinoic acid [31,32]. ALDH also plays a role in reactive oxygen species (ROS) scavenging and thereby reducing oxidative stress in stem cells [33] (Figure 1). 

In cancer cells, ALDH contributes to chemoresistance via different mechanisms [34]. ALDH isoforms, ALDH1A1, and ALDH3A1 are both involved in the metabolism of the cancer drug cyclophosphamide, metabolizing the active compound to a less active form and contributing to drug resistance [35]. When combined with cyclophosphamide in ALDH3A1^high^ cell lines, ALDH3A1 inhibitors have been shown to increase sensitivity to the mafosphamide (cyclophosphamide analog) [34]. Another mechanism by which ALDH protects cancer cells is by reducing ROS-mediated oxidative stress [36] (Figure 1). As a consequence of this, ALDH^high^ CSCs have a lower baseline ROS level and oxidative damage than the ALDH^low^ counterparts [33].

Clinically, high ALDH expression is associated with poor outcomes in several gynecologic malignancies, including ovarian cancer (OC) [17,37,38,39,40], endometrial [41], and cervical cancer (CC) [42,43], as well as other solid tumors including breast [44,45,46], lung adenocarcinoma [47], rectal [48], esophageal squamous adenocarcinoma [49], gastric [50], colorectal [51], prostate [52], and neuroblastoma [53]. To our knowledge, there are no published reports correlating ALDH and prognosis in vulvar or vaginal cancer. Of the 19 ALDH isoforms, ALDH1 is the primary isoform implicated in CSCs of solid tumors [54]. The advent of the Aldefluor assay has stimulated research on CSCs expressing high ALDH. Briefly, the Aldefluor assay can be used to detect cells expressing high levels of ALDH. The Aldefluor reagent Bodipy- aminoacetaldehyde (BAAA) is converted into BODIPY-aminoacetate (BAA), in the presence of ALDH, and retained inside the cells. ALDH activity of the cells is directly proportional to the fluorescence intensity [55]. The assay can detect nine ALDH isoforms, with ALDH1 as the predominant isoform contributing to ALDH^high^ cells [56]. These provide a strong rationale for targeting ALDH to eliminate CSCs in gynecologic malignancies. A recent review by Dinavahi et al. elegantly highlights the inhibitors developed to target ALDH from a pharmacologic perspective in different cancer types [57].

In this review, we briefly discuss the gynecologic malignancies affecting women worldwide and review the findings about ALDH in relation to the gynecologic CSCs. We assess the agents targeting ALDH directly or indirectly in the different gynecologic cancers. Finally, we discuss current challenges associated with targeting ALDH in CSCs in gynecologic cancers.

## 2. Gynecologic Malignancies: An Overview

**Figure 2:**
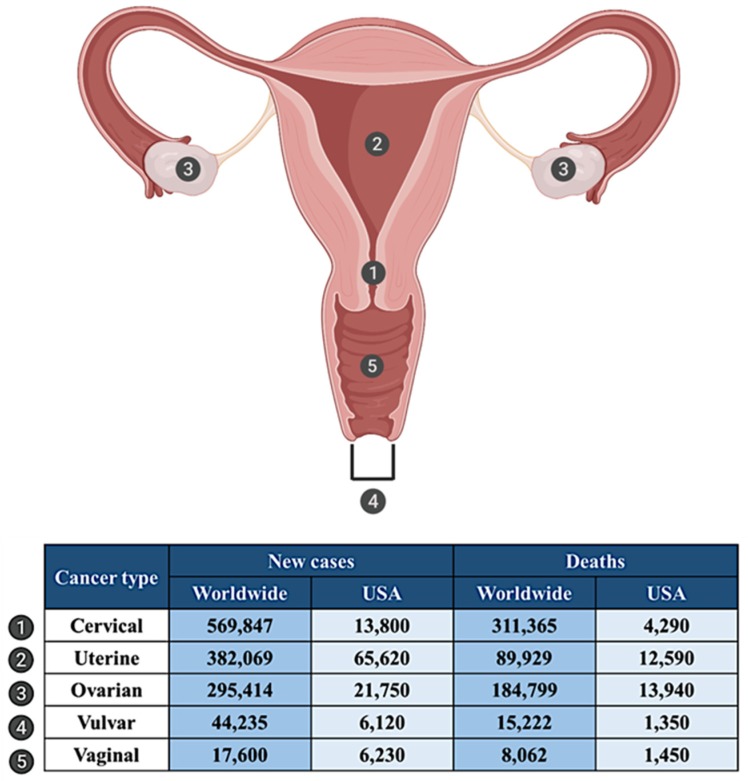
Site of gynecologic cancers in the female reproductive tract and statistics for incidence and mortality worldwide (2018) [58] and in USA [60].

Gynecologic malignancies are cancers that originate in the female reproductive system. Each year over 1.3 million women are diagnosed with gynecologic cancers worldwide, and over 600,000 deaths are caused due to gynecologic cancers [58]. There are five types of gynecologic cancers based on the site of origin, namely ovarian, cervical, uterine, vaginal, and vulvar (Figure 2). Although these cancers are commonly grouped, each has unique features, symptoms, and treatment options [59].

### 2.1. Cervical Cancer 

Cervical cancer (CC) is the deadliest of the gynecologic cancers, causing more deaths worldwide compared to all other cancers of the female reproductive system [58]. In 2018, 569,847 women were diagnosed with CC worldwide. CC claimed 311,365 lives [58] (Figure 2). CC is divided into two main subtypes: squamous cell carcinoma (SCC) and adenocarcinoma. SCC originates from the squamous cells lining the ectocervix (part of the cervix extending outwards to the vagina) and is the most prevalent type, accounting for about 80% of CC. 

### 2.2. Uterine Cancer

According to the statistics by the International Agency for Research on Cancer (IARC), approximately 382,069 new cases of cancer of the uterus (uterine body or corpus) were diagnosed in 2018, and about 89,929 women died from uterine cancer [58] (Figure 2). Uterine cancer is classified into two main types: endometrial cancer (EC) and uterine carcinosarcoma. EC are further divided into type I and type II malignancies [61]. Type I cancers are the most common and affect both pre- and postmenopausal women and are generally low grade, well-differentiated tumors of endometrioid histology [61]. Most EC is diagnosed at initial stages, making it possible to treat the disease with cytoreductive surgery [62]. However, when diagnosed with extrauterine spread, the relapse rate is high [62].

### 2.3. Ovarian Cancer

Ovarian cancer (OC) is a deadly gynecologic malignancy and is the second leading cause of death due to gynecologic cancers worldwide [58]. About 295,414 women were diagnosed with OC in 2018, and the disease caused about 184,799 deaths globally [58] (Figure 2). The most common type of OC is epithelial OC, which is categorized into four histological subtypes, namely serous, clear cell, endometrioid, and mucinous carcinomas [63]. Of these, the most significant contributor to high mortality and reduced survival rate is high grade serous ovarian cancer (HGSOC) [64]. In most women, the diagnosis is made only at advanced stages, after the tumor has spread beyond the ovary [64]. HGSOC is initially highly responsive to platinum or taxane-based chemotherapy; however, more than 80% of patients experience disease relapse and progression. Chemoresistant HGSOC is uniformly fatal [64]. The 5-year survival rate for HGSOC is only about 30% and has not improved in the last several decades [65].

### 2.4. Vulvar Cancer

Vulvar cancer is a rare malignancy of the vulva, accounting for about 6% of cancers of female reproductive organs and less than 1% of all cancers in women [60]. Globally, 44,235 new cases of vulvar cancer were diagnosed in 2018, and about 15,222 women died due to vulvar cancer [58] (Figure 2). Vulvar cancer includes cancers of the inner and outer lips of the vagina, the clitoris, and the opening of the vagina and vaginal glands. The main subtypes of vulvar cancer are SCC, adenocarcinoma, sarcoma, and basal cell carcinoma, amongst which the most prevalent is SCC [66]. Surgery is the primary mode of treatment for vulvar cancer, followed by radiation or chemotherapy [67]. 

### 2.5. Vaginal Cancer

Vaginal cancer is an uncommon cancer type and comprises about 1% of all gynecologic malignancies [60]. According to the IARC, 17,600 cases of vaginal cancer were diagnosed in 2018, and the disease caused about 8062 deaths worldwide [58] (Figure 2). Approximately 85% of vaginal cancers are malignancies in the squamous cell lining. At an early stage, this cancer is curable by surgery and radiation. However, only radiation therapy is known to be effective in advanced stage vaginal cancer [68].

## 3. ALDH in Gynecologic Cancers

### 3.1. ALDH and Cervical Cancer

In tissue specimens from patients with cervical SCC or cervical intraepithelial neoplasia (CIN) II-III, high ALDH expression was observed immunohistochemically [43]. Interestingly, peripheral blood (plasma) samples from the same patients had increased ALDH1A1 expression when compared with samples from healthy patients [43]. In patients with invasive SCC, ALDH1 expression correlated with lymph nodal metastasis and disease recurrence [69]. These data indicate that ALDH1 can be used as a reliable biomarker for the identification of cervical CSCs [70]. The ALDH^high^ cells isolated from these cancers showed high gene and protein expression of stemness transcription factors Nanog, sex determining region Y-box2 (Sox2), octamer-binding transcription factor (Oct4), and twist-related protein 1 (Twist1) [43]. However, the exact mechanism by which ALDH regulates stemness in cervical cancer remains incompletely understood.

### 3.2. ALDH and Uterine Cancer

In EC, the most prevalent type of uterine cancer, altered stemness related pathways, including Wnt and β-catenin, support a role for CSCs [71]. Furthermore, ALDH^high^ subpopulation of cells has been demonstrated to be CSCs in EC [71]. The ALDH^high^ cells isolated from primary endometrial tumors were highly tumorigenic and resistant to chemotherapeutic drugs and showed increased invasive ability compared to the ALDH^low^ cells [72]. 

In patients with uterine endometrioid carcinosarcoma, high ALDH1 expression predicted poor prognosis, lymphatic invasion, recurrence, and low overall survival [73]. ALDH^high^ CSCs cells have distinct stem-like properties, such as high expression of stem-cell markers BMI1, HEY1, HES1, and adhesive molecule CD44 [41,74,75]; in addition, reduced expression of differentiation markers, enhanced migration, high tumorigenicity, and self- renewal ability were reported [76]. When EC cells were sorted using flow cytometry and cultured in vitro, ALDH^high^ cells yielded both ALDH^high^ and ALDH^low^ cells, whereas ALDH^low^ cells only yielded ALDH^low^ cells [72]. These results demonstrated that endometrial CSCs divide asymmetrically, in agreement with the CSC hypothesis. Furthermore, when injected into mice subcutaneously, ALDH^high^ endometrial cells formed larger tumors more rapidly than the ALDH^low^ cells [72], demonstrating that CSCs were able to repopulate the entire endometrial tumor mass. Based on these studies ALDH1 serves as both a marker for identifying endometrial CSCs and a therapeutic target, based in its functional importance in the disease.

### 3.3. ALDH and Ovarian Cancer

Cancer relapse after surgery and chemotherapy is common in OC, and CSCs are strongly associated with OC relapse [77]. ALDH^high^ cells are widely accepted as CSCs in OC, as demonstrated by us and others [17,37,38,39,78,79,80,81,82,83]. ALDH^high^ ovarian cancer stem cells (OCSCs) exhibit classic stem cell characteristics, such as being highly chemoresistant and enriched in residual xenografts after platinum therapy [78,79,83]. Upregulation of stemness genes such as Sox2, Kruppel like factor 4 (Klf4), Nanog, and downregulation of differentiation genes such as homeobox A10 (HOXA10) and homeobox A11 (HOXA11), were reported in ALDH^high^ cells [83]. ALDH^high^ cells demonstrated enhanced ability to form spheroids in low attachment conditions in vitro [75,80]. 

In OCSCs, of the 19 ALDH isoforms, ALDH1A1 was highly correlated with chemotherapy resistance [78,79,81,82,84,85]. Expression of ALDH1A1 was 100-fold higher in OC cells selected for taxane-resistance in vitro, and ALDH1A1 knockdown sensitized the resistant cells to chemotherapy [82]. ALDH1A1 expression was higher in residual tumors after the first round of chemotherapy compared to tumors from untreated patients [86], demonstrating enrichment of OCSC post-treatment. In addition to ALDH1A1, CD133 serves as a robust marker for OCSC when used in combination with ALDH [17]. In this regard, Silva et al. observed that as low as 11 ALDH and CD133 double-positive cells resulted in tumor induction in mice [17]. Moreover, in tumors harvested during debulking surgeries, ALDH^high^CD133+ cells correlated with reduced disease-free and overall survival in OC patients [17]. ALDH^high^ cells were highly metastatic with enhanced invasive ability and were resistant to apoptosis [87]. These data provide a strong rationale for targeting ALDH^high^ cells in OC [37].

### 3.4. ALDH and Vulvar Cancer

Vulvar cancer is an uncommon type of tumor in women. To date, one study on ALDH in this gynecologic cancer has been reported. ALDH1 expression in vulvar squamous cell carcinoma, normal vulvar epithelium, and stromal tissues in a cohort of 154 patients was studied [88]. Based on clinicopathological studies, high ALDH1 expression correlated with favorable prognosis and can be considered a potential marker for differentiated vulvar cells [88]. However, this report is contradictory to the correlation of ALDH with poor prognosis in other gynecologic cancers, suggesting that ALDH expression could be a tissue-specific marker for CSCs. 

## 4. ALDH-Targeted Therapies for Gynecologic Cancers

### 4.1. Agents Directly Targeting ALDH

The identification of ALDH^high^ cells as CSCs, first reported in breast cancer tissue [89], is widely accepted as a CSC marker in most other tumors [90,91,92,93,94,95,96,97,98,99]. Landen et al. was the first to demonstrate proof-of-concept for targeting ALDH^high^ cells in a gynecologic cancer [79]. In ovarian CSC, use of small interfering ribonucleic acid (siRNA) to silence ALDH1A1 in an orthotopic mouse model sensitized both platinum and taxane resistant OC cell lines to chemotherapy [79]. In this section, we discuss the inhibitors used to target ALDH in gynecologic malignancies (Table 2).

#### 4.1.1. Disulfiram

Disulfiram (DSF) is an orally bioavailable ALDH inhibitor, well known for its effectiveness in the treatment of alcohol addiction [113,114]. Though DSF is primarily an inhibitor for ALDH2, DSF also inhibits ALDH1 isozymes, which are overexpressed in CSCs, providing the rationale for repurposing DSF as an anti-cancer drug. Anti-neoplastic activities of DSF in several malignancies have been described [115,116,117,118,119,120]. DSF works by elevating intracellular ROS levels, thus driving the CSCs towards apoptosis [121]. DSF/Copper complex was shown to target ALDH1A1 and inhibit cancer recurrence primarily driven by ALDH^high^ CSCs [100]. In OC, DSF exhibited cytotoxicity comparable to cisplatin, while having a limited effect on the viability of non-neoplastic cells. Cell death due to DSF was partly due to programmed cell death, and an additive effect, when combined with chemotherapy, was observed [101] (Table 2). Interestingly, DSF depleted CD44+ OCSCs in vitro but not CD133+ cells in vitro. However, DSF did not show significant activity in vivo [101]. In another study, DSF induced cell death in OC by promoting a pro-oxidative intracellular environment, causing irreversible damage to the cancer cells within a few hours of treatment via the induction of heat shock proteins [102].

In the treatment of localized CC, DSF-loaded thermoplastic vaginal rings were shown to be effective in vitro [103] (Table 2). The vaginal rings are a minimally invasive treatment option as well as require low drug concentration compared to oral delivery. However, vaginal rings require further in vivo and clinical validation and are currently not a therapeutic option [103]. Presently, there are no ongoing clinical trials using DSF to treat gynecologic malignancies. Limited in vivo efficacy of DSF and lack of selectivity has limited its use as an ALDH inhibitor and CSC-targeted agent. To our knowledge, there are no published reports on the use of DSF in other gynecologic malignancies.

#### 4.1.2. 4-Diethylaminobenzaldehyde

4-Diethylaminobenzaldehyde (DEAB) is an ALDH inhibitor and a slow competitive substrate for the enzyme [122]. DEAB has been extensively used to inhibit retinoic acid (RA) synthesis in studying the role of retinoic acid in embryogenesis [123]. DEAB is commonly used as a negative control in the Aldefluor assay to detect ALDH^high^ CSCs [44]. In a HGSOC study, high dose DEAB treatment preferentially depleted CD133+ ovarian CSCs [80] (Table 1). In chemoresistant OC cell lines, DEAB treatment significantly inhibited P-glycoprotein (P-gp) protein and breast cancer resistance protein (BRCP) transcript and protein, re-sensitizing the cell lines to chemotherapy [82]. Similarly, DEAB effectively inhibited the proliferation of spheroids generated from patient-derived endometrial CSCs [104] (Table 2). However, it is also important to note that the selectivity of DEAB for different ALDH isoforms is low. DEAB acts as a substrate with the highest selectivity for the isoform ALDH3A1 expressed in stem cell populations in various tumor tissues [105]. DEAB is a substrate for ALDH1A1 (albeit very slow) [105]. No published reports of DEAB being used to target ALDH in other gynecologic cancers were found.

#### 4.1.3. All-Trans Retinoic Acid (ATRA)

ATRA is an active metabolite of vitamin A and involved in many crucial signaling pathways such as embryonic development, immune system function, reproduction, and epithelial integrity [124,125,126]. ATRA is commonly used as a differentiation agent in stem cell research [127,128], and several studies have reported the use of ATRA as a differentiation agent to inhibit cancer cell growth by altering the cell cycle progression [129,130,131] and to target CSCs tumorspheres in various malignancies both in vitro and in vivo [132,133,134,135,136]. In OC, ATRA reduced ALDH1 expression and suppressed in vitro tumorsphere formation, cell migration, and invasion and in vivo tumorigenesis [106] (Table 2). The mechanism of action of ATRA was shown to be mediated through the abrogation of ALDH1/FoxM1/Notch1 signaling in OCSCs [106]. In another study in OC cell lines, ATRA-mediated inhibition of ALDH1A1 activity led to the re-sensitization of the resistant cells to chemotherapeutics, by downregulating the expression of drug transporters P-gp and BCRP on the cell surface [82]. ATRA suppresses ALDH1 expression by inhibiting nuclear factor erythroid-2-related factor 2 (Nrf-2), a transcription factor associated with chemoresistance and cancer progression in OC [107]. Inhibition of ALDH1A1 by ATRA led to the inhibition of the CSC-like properties and the expression of drug efflux transporters, and p62, along with CSC markers in ALDH^high^ cells [107]. In CC CSCs, ATRA reduced the percentage of ALDH^high^ cells by targeting the expression of ALDH1A1 and ALDH1A3 [108] (Table 2). There are no published reports of ATRA being used as a therapeutic agent to target CSCs in the other gynecologic malignancies.

#### 4.1.4. NCT-501, NCT-505, and NCT-506

To identify a specific ALDH1A1 inhibitor, Yang et al. performed a systematic medicinal chemistry optimization of theophylline-based compounds through a miniaturized 1536-well Aldefluor assay screen [137]. This screen identified compound NCT-501 as a specific ALDH1A1 inhibitor with enhanced ADME properties. NCT-501 exhibited exceptional selectivity over other ALDH isozymes (ALDH1B1, ALDH2, and ALDH3A1) (Table 2). NCT-501 is a reversible inhibitor with good in vivo pharmacological exposure by intraperitoneal administration (i.p.); however, further development is necessary in order to make the drug orally bioavailable [85]. In OC cells, ALDH1A1 inhibition by NCT-501 abrogated CSC expansion induced by the silencing of a tumor suppressor DNA damage-binding protein (DDB2) [109]. Treatment with NCT-501 inhibited ALDH activity without significant cytotoxicity and reduced the de-differentiation of non-CSC into CSC significantly [81]. In endometrial CSCs, NCT-501 caused significant inhibition of ALDH activity, reduced spheroid cells, and caused selective cell death in ALDH^high^ cells [110].

Medicinal chemistry optimization also led to the discovery of newly designed ALDH1A1 selective inhibitors NCT-505 and NCT-506 [138] (Table 2). These analogs showed target engagement in a cellular thermal shift assay (CETSA) in vitro. In a cellular context, the inhibitors inhibited spheroid formation in an OC cell line and re-sensitized paclitaxel-resistant OC cells to cytotoxicity. These lead compounds also exhibited enhanced selectivity over other ALDH isozymes. Pharmacokinetic studies have shown in vivo drug exposure for NCT-505 and NCT-506 [108], establishing the potential for proof-of-concept experiments to better understand ALDH1A1 regulation in CSCs in vivo.

#### 4.1.5. CM037 

Toward the objective of identifying a specific ALDH1A1 inhibitor, Morgan et al. developed a high-throughput screening (HTS) platform using an in vitro NAD+ independent esterase assay. Using this HTS platform, 64,000 compounds were screened and a potent, highly selective, and novel ALDH1A1 inhibitor CM037 (A37) was identified [111] (Table 2). In OC cells, Matei and Hurley et al. demonstrated that CM037 significantly inhibited tumorsphere formation and CSC viability [84]. In a subsequent study by the same group, CM037 treatment promoted DNA damage in OC cells with a corresponding increase in DNA damage response genes. CM037 inhibited ALDH1A1, resulting in the accumulation of intracellular ROS, DNA damage, and apoptosis [84]. In EC cells, Mori el al. demonstrated that CM037 treatment inhibited spheroid formation and preferential death of ALDH^high^ cells [110]. Thus, CM037 is a potential therapeutic to target ALDH1A1 in CSCs in OC and EC; however, further modifications in the structure are required to improve the in vivo stability and bioavailability of the inhibitor. No published reports on CM037 in other gynecologic malignancies could be found in the literature. 

#### 4.1.6. 13g, 13h

A (HTS) approach by Hurley et al. identified CM039, a lead compound with high selectivity towards ALDH1A1 [139]. A co-crystal structure of CM039 with ALDH1A1 yielded two compounds with high selectivity towards ALDH1A1 over ALDH2 isoform, named 13g and 13h [112] (Table 2). Both compounds significantly reduced the proliferation of ALDH^high^CD133+ OCSC population in HGSOC cell lines and showed excellent in vivo efficacy (i.p. administration) [112] and 13h synergized with cisplatin in a patient-derived OC spheroid model [112].

#### 4.1.7. 673A

673A, an analog of DEAB, is a pan-ALDH1A inhibitor. 673A can inhibit ALDH1A1, ALDH1A2, and ALDH1A3, with high selectivity over ALDH2 or ALDH3. 673A preferentially killed CD133 + CSCs by induction of necroptosis and exhibited synergy with chemotherapy in reducing tumor initiation and promoting tumor eradication in vivo [80] (Table 2). This study confirmed the role of ALDH1A family enzymes in chemoresistance and strengthened the hypothesis that targeting ALDH family members can improve outcomes in patients with HGSOC.

### 4.2. Agents Indirectly Targeting ALDH

Indirect targeting involves inhibition of targets upstream regulators of ALDH, thus blocking the expression of ALDH in the cells. The studies described below have taken this approach (Table 3).

#### 4.2.1. JQ1

JQ1 is a thienotriazolodiazepine and a potent inhibitor that targets bromodomain-containing protein 4 (BRD4), a transcriptional regulator for global gene expression patterns [144]. JQ1 inhibited ALDH activity in epithelial OC cells by acting on the super-enhancer of the ALDH1A1 gene and blocked the growth of cisplatin-resistant OC cells in vitro [140] (Table 3). In an orthotopic mouse model, Zhang and co-workers further demonstrated that JQ1 in combination with cisplatin improved the survival of tumor-bearing mice [140], providing compelling rationale for the use of JQ1 as a potential therapeutic agent to target OCSCs and perhaps CSCs in other gynecologic malignancies.

#### 4.2.2. Anti-EMP2 IgG1

In EC patients, epithelial membrane protein-2 (EMP2) correlated with disease progression and poor survival [145]. Treatment with the Anti-EMP2 IgG1 antibody inhibited the expression and activity of ALDH and correspondingly reduced both primary and secondary tumor load [141] (Table 3). However, further research is required to improve drug delivery mechanisms to administer the antibody-based treatment to patients. 

#### 4.2.3. Peptide Nucleic Acid 

Non-protein coding RNAs consisting of small (<200 nucleotides) and long (>200 nucleotides) non-coding RNAs (ncRNAs) constitute a large proportion of transcripts in the human genome. Specifically, aberrant expression of long non-coding RNA (lncRNA) HOX antisense intergenic RNA (HOTAIR) is associated with cancer progression and metastasis in several cancer types [146,147,148]. Our lab has previously reported that HOTAIR is highly expressed in chemo-resistant OC [149] and in ALDH^high^ OCSCs [142]. Targeting HOTAIR with peptide nucleic acid (PNA) inhibited ALDH1A1 levels in OCSCs in vitro and in vivo [142] (Table 3), representing a novel strategy to target lncRNAs and subsequently ALDH1A1 in gynecologic CSCs.

#### 4.2.4. miR-23b

Micro-RNAs (miR) are short ncRNAs involved in pathogenesis of various malignancies including CC [150]. miR-23b was downregulated in cervical CSCs derived from tumorspheres and direct binding of miR-23b to the 3’ untranslated region (UTR) suppressed translation of ALDH1A1 protein [143] (Table 3). In addition, overexpression of miR-23b increased the sensitivity of cervical CSCs to platinum-based chemotherapy [143], supporting the potential of miR-23b as an indirect approach to target ALDH1A1 in cervical CSCs.

### 4.3. Combination Therapies using ALDH Inhibitors

Several studies in OC have demonstrated the therapeutic potential of combining ALDH inhibitors with chemotherapy. siRNA-mediated gene silencing of ALDH1A1 sensitized cisplatin and paclitaxel-resistant OC cell lines to chemotherapy and significantly inhibited tumorigenesis in mice compared to chemotherapy alone [79]. Treatment with an ALDH1A1 inhibitor CM037 sensitized OC spheroids to cisplatin treatment [78]. ALDH1A family inhibitor 673A synergized with cisplatin treatment, resulting in a 30-fold reduction in cell numbers in chemotherapy-resistant OC cells compared to either therapy alone [80] and was also shown to synergize with cisplatin to inhibit ovarian CSC proliferation [112]. Auranofin, an inhibitor of the cellular thioredoxin system, enhanced the cytotoxic effect of DSF in OC cells [102]. This is an excellent example of drug repositioning where a combination of anti-alcoholic disulfiram and the anti-rheumatic auranofin may be a potential therapeutic option for recurrent OC [102]. Finally, pretreatment with a combination of ATRA and DEAB re-sensitized ALDH^high^ OCSCs to chemotherapeutic drugs paclitaxel and topotecan [82]. In EC patient-derived spheroids, a combination of paclitaxel and ALDH inhibitor DSF chemotherapy demonstrated synergistic inhibition of cancer progression in vivo and in vitro [110]. Reports on combination treatments using ALDH inhibitors in the other gynecologic malignancies were not apparent; however, published studies in OC and EC provide strong rationale for the treatment of chemo-resistant gynecologic malignancies in general with a combination of chemotherapy and ALDH inhibitors to achieve a better outcome in patients. 

## 5. Challenges and Future Directions

A challenging aspect of using ALDH inhibitors to target CSCs is the broad expression of the enzyme family in healthy tissues, especially in the liver and kidney [151]. Until recently, ALDH1A1 was thought to be primarily responsible for the stemness phenotype. However, recent reports suggest that other isoforms, including ALDH1A3, ALDH7A1, and ALDH3A1 [152,153,154], contribute to stemness, demonstrating the need to develop additional isoform-specific inhibitors. Another challenge is in vivo activity which explains the lack of ALDH inhibitors in ongoing clinical trials for gynecologic cancers. To date, 673A is the only isoform-selective compound used in vivo [80]. Even with several ALDH inhibitors being developed recently [57], it is essential to focus efforts on developing efficacious and bioavailable ALDH inhibitors to fully understand the role of ALDH in CSC regulation in vivo. Lastly, even though the use of specific inhibitors to target CSCs may eliminate the resistant population, it is unlikely that ALDH inhibitors will be effective as single agents. warranting further exploration of ALDH inhibitors in combination with cytotoxic or targeted therapies in gynecologic cancers and other malignancies.

## 6. Conclusions

Research on ALDH in CSCs over the past several decades has dramatically advanced the understanding of biological processes involved in tumorigenesis and chemoresistance. Of the several markers implicated in CSCs, ALDH is the most robust and has the advantage of having a functional role in the maintenance and protection of CSCs. The development of new inhibitors and repurposing of several ALDH inhibitors are promising approaches for targeting and eradicating CSCs. However, in different malignancies, including gynecologic cancers, isoform specific ALDH inhibitors are needed to target the full spectrum of CSCs. In addition, ALDH inhibitors with improved pharmacokinetic properties are needed for use in the clinic for patients with gynecologic and other malignancies.

## Figures and Tables

**Table 1:** Cancer stem cells reported in gynecologic malignancies.

Gynecologic Malignancy	Cancer Stem Cells	References
Cervical cancer	Reported in literature	[8,9,10]
Uterine cancer	Reported in literature	[11,12,13,14]
Ovarian cancer	Reported in literature	[15,16,17,18,19]
Vulvar cancer	Reported in literature	[20]
Vaginal cancer	No published reports	-

**Table 2:** ALDH inhibitors in gynecologic malignancies.

Compound	ALDH Isoform Specificity	Gynecologic Malignancy	Preclinical Studies	References
Disulfiram	ALDH2, ALDH1	Ovarian Cervical	Orally bioavailable. Limited in vivo efficacy in ovarian cancer mouse model	[100,101,102,103]
DEAB	ALDH1A1, ALDH1A2, ALDH1A3, ALDH1B1, ALDH2, ALDH3A1, ALDH5A1	Ovarian Endometrial	No in vivo studies found	[80,82,104,105]
ATRA	ALDH1, ALDH1A1, ALDH1A3	Ovarian Cervical	Reduced in vivo tumorigenesis in ovarian cancer	[106,107,108]
NCT 501	ALDH1A1	Ovarian Endometrial	Halted tumor re-growth in orthotropic ovarian cancer xenograft model	[85,109,110]
NCT-505 NCT-506	ALDH1A1	Ovarian	Orally bioavailable No weight loss or mortality in pilot toxicity studies	[108]
CM037 (A37)	ALDH1A1	Ovarian Endometrial	Ineffective in vivo likely due to low aqueous solubility Non-toxic to mice at 20 mg/kg	[84,110,111]
13g 13h	ALDH1A	Ovarian	Showed excellent in vivo efficacy on i.p. administration in OC mouse model	[112]
673A	ALDH1A1, ALDH1A2, ALDH1A3	Ovarian	Highly synergistic with chemotherapy in reducing tumor initiation and increasing tumor eradication	[80]

**Table 3:** Agents indirectly targeting ALDH in gynecologic malignancies.

Therapeutic Agent	Target	Gynecologic Malignancy	In Vivo Studies Reported	References
JQ1	BRD4	Ovarian cancer	JQ1 in combination with cisplatin improved survival of OC bearing mice in an orthotopic model	[140]
Anti-EMP2 IgG1	EMP2	Endometrial cancer	Reduces CSCs and secondary tumor formation in mice	[141]
PNA	HOTAIR	Ovarian cancer	Reduced tumor formation and improved survival in mice with platinum-resistant ovarian tumor xenografts	[142]
miR-23b	3’UTR of ALDH1A1	Cervical cancer	No in vivo studies reported	[143]

## Abbreviations

CSC	cancer stem cells
ALDH	aldehyde dehydrogenases
RA	retinoic acid
ROS	reactive oxygen species
ABC	adenosine triphosphate binding cassette
ATM	ataxia-telangiectasia-mutated
ATR	ataxia telangiectasia and rad3-related
Chk1	checkpoint kinase 1
PARP1	poly-ADP-ribose polymerase 1
BAAA	Bodipy-aminoacetaldehyde
BAA	Bodipy-aminoacetate
CC	Cervical cancer
SCC	squamous cell carcinoma
IARC	International Agency for Research on Cancer
EC	endometrial cancer
OC	Ovarian cancer
HGSOC	high grade serous ovarian cancer
CIN	cervical intraepithelial neoplasia
Oct4	octamer-binding transcription factor 4
Sox2	sex determining region Y-box 2
Twist 1	Twist-related protein 1
Hey 1	Hes related family BHLH transcription factor with YRPW motif 1
Klf4	Kruppel like factor 4
HOXA10	Homeobox A10
HOXA11	Homeobox A11
siRNA	small interfering ribonucleic acid
DSF	Disulfiram
DEAB	4-Diethylaminobenzaldehyde
P-gp	P-glycoprotein
BRCP	breast cancer resistance protein
HTS	high-throughput screening
ATRA	All-trans retinoic acid
Nrf-2	nuclear factor erythroid-2-related factor 2
i.p.	intraperitoneal administration
DDB2	DNA damage-binding protein
CETSA	cellular thermal shift assay
BRD4	Bromodomain-containing protein 4
EMP2	epithelial membrane protein-2
lncRNA	long non-coding RNA
HOTAIR	HOX antisense intergenic RNA
PNA	peptide nucleic acid
miR	micro-RNAs
UTR	untranslated region
